# A model for malaria elimination based on learnings from the Malaria Elimination Demonstration Project, Mandla district, Madhya Pradesh

**DOI:** 10.1186/s12936-021-03607-3

**Published:** 2021-02-16

**Authors:** Harsh Rajvanshi, Praveen K. Bharti, Sekh Nisar, Himanshu Jayswar, Ashok K. Mishra, Ravendra K. Sharma, Kalyan B. Saha, Man Mohan Shukla, Suman L. Wattal, Aparup Das, Harpreet Kaur, Anupkumar R. Anvikar, Azadar Khan, Nilima Kshirsagar, Aditya P. Dash, Altaf A. Lal

**Affiliations:** 1 Malaria Elimination Demonstration Project, Mandla, Madhya Pradesh India; 2 Indian Council of Medical Research-National Institute of Research in Tribal Health (ICMR- NIRTH), Jabalpur, Madhya Pradesh India; 3 Directorate of Health Services, Government of Madhya Pradesh, Bhopal, India; 4 National Vector Borne Disease Control Programme, Ministry of Health and Family Welfare, New Delhi, India; 5 Indian Council of Medical Research, Department of Health Research, Ministry of Health and Family Welfare, New Delhi, India; 6 Indian Council of Medical Research-National Institute of Malaria Research (ICMR-NIMR), New Delhi, India; 7 Foundation for Disease Elimination and Control of India, Mumbai, Maharashtra India; 8 Asian Institute of Public Health University, Odisha, India

**Keywords:** Malaria model, Malaria elimination, Mandla, National malaria elimination

## Abstract

**Background:**

Malaria Elimination Demonstration Project (MEDP) was started as a Public-Private-Partnership between the Indian Council of Medical Research through National Institute of Research in Tribal Health, Govt. of Madhya Pradesh and Foundation of Disease Elimination and Control of India, which is a Corporate Social Responsibility (CSR) initiative of the Sun Pharmaceutical Industries Limited. The project’s goal was to demonstrate that malaria can be eliminated from a high malaria endemic district along with prevention of re-establishment of malaria and to develop a model for malaria elimination using the lessons learned and knowledge acquired from the demonstration project.

**Methods:**

The project employed tested protocols of robust surveillance, case management, vector control, and capacity building through continuous evaluation and training.  The model was developed using the learnings from the operational plan, surveillance and case management, monitoring and feedback, entomological investigations and vector control, IEC and capacity building, supply chain management, mobile application (SOCH), and independent reviews of MEDP.

**Results:**

The MEDP has been operational since April 2017 with field operations from August 2017, and has observed: (1) reduction in indigenous cases of malaria by about 91 %; (2) need for training and capacity building of field staff for diagnosis and treatment of malaria; (3) need for improvement insecticide spraying and for distribution and usage of bed-nets; (4) need for robust surveillance system that captures and documents information on febrile cases, RDT positive individuals, and treatments provided; (5) need for effective supervision of field staff based on advance tour plan; (6) accountability and controls from the highest level to field workers; and (7) need for context-specific IEC.

**Conclusions:**

Malaria elimination is a high-priority public health goal of the Indian Government with a committed deadline of 2030. In order to achieve this goal, built-in systems of accountability, ownership, effective management, operational, technical, and financial controls will be crucial components for malaria elimination in India. This manuscript presents a model for malaria elimination with district as an operational unit, which may be considered for malaria elimination in India and other countries with similar geography, topography, climate, endemicity, health infrastructure, and socio-economic characteristics.

## Background

In the year 2019, there were an estimated 229 million cases worldwide. Twenty-nine countries contributed to 95 % of the global malaria burden with 94 % of the cases being contributed by the WHO African Region. Although the major burden was borne by African nations, progress was noticed in other parts of the world. Paraguay has been certified as malaria free, Algeria, Argentina and Uzbekistan have made formal requests for the certification, and in 2018 and 2019–China and El Salvador reported zero indigenous cases, respectively. Sri Lanka is malaria free and many countries in the Asia Pacific region are on track to eliminate malaria by 2030. India has the highest number of malaria cases (2 % of global cases) and deaths (2 % of malarial deaths) outside of the African sub-continent [1].

**Fig. 1:**
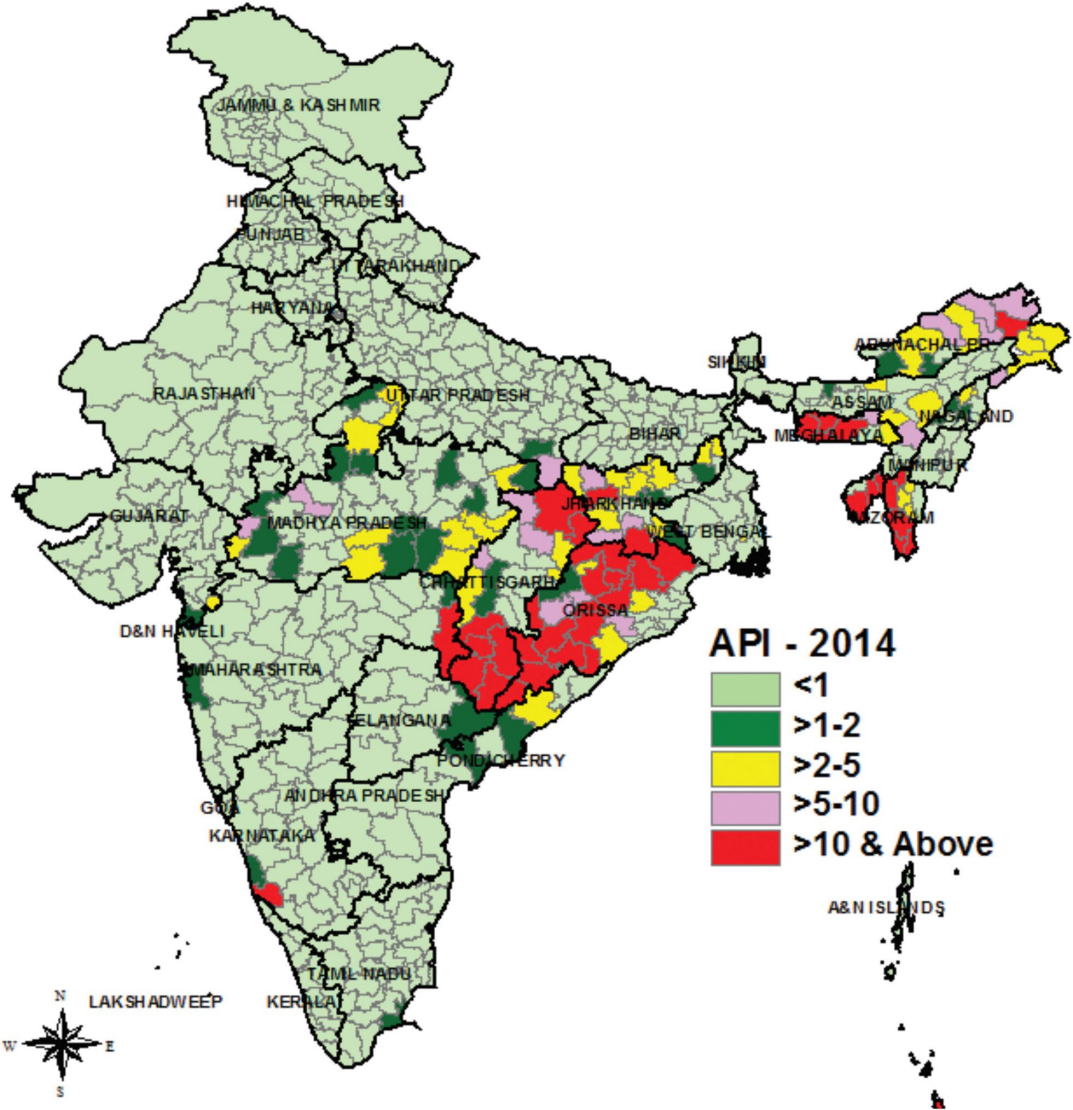
Map of India showing the malaria distribution. States of Odisha, Chattisgarh, Madhya Pradesh, and the north eastern states are most affected by malaria. (Source: NVBDCP)

More than 80 % of tribal population of India resides in ten states viz. Madhya Pradesh, Odisha, Rajasthan, Maharashtra, Jharkhand, Andhra Pradesh, Chattisgarh, West Bengal, Gujarat and Karnataka. Out of which, Madhya Pradesh and Maharashtra constitute one-fourth of total tribal population. Similarly, more than 80 % malaria cases were reported from these tribal states [2]. Figure 1 shows the status of malaria in India in 2014 [3].

The National Vector Borne Disease Control Programme’s (NVBDCP) commitment to end malaria aligns with rest of the world and responds to the commitment for malaria elimination by 2030. The Strategic Plan for Malaria Control in India 2012–2017: A Five-year Strategic Plan was followed by development of the National Framework for Malaria Elimination in India 2016–2030, which made the target as ‘elimination’ and not just ‘control’ for the entire country. The plan provided details of technical and operational element required to achieve malaria elimination by 2030 [3, 4].

Complementing the national malaria elimination programme, Sun Pharmaceutical Industries Ltd. established the Foundation for Disease Elimination and Control (FDEC) of India (a not-for-profit entity) as part of its Corporate Social Responsibility (CSR) initiative. The FDEC entered into a public-private partnership with the Indian Council of Medical Research (ICMR) through the National Institute of Research in Tribal Health (ICMR-NIRTH) and Government of Madhya Pradesh, with a goal to demonstrate that malaria can be eliminated from a defined area of high-endemicity that posed demographic and epidemiologic challenges. The Malaria Elimination Demonstration Project (MEDP) has shown about 91 % reduction in indigenous cases of malaria in all 1233 villages of the district by employing robust surveillance, case management, vector control, IEC/BCC, and capacity building.

This paper presents models for malaria elimination, which may be considered for adoption in the entire state of Madhya Pradesh and rest of the country with similar geography, topography, climate, endemicity, health infrastructure, and socio-economic characteristics.

## The Mandla Malaria Elimination Demonstration Project (MEDP)

The objective of the project is to demonstrate that malaria can be eliminated from all 1233 villages of district Mandla, which was a high endemic district with varying levels of API at the beginning of the project. As of 2020, the population of the district is approximately 1.15 million. Mandla offers varying complexities of demographic, epidemiology and access to health care (multiplicity of health care providers in urban and rural areas).

## Methods

The National Vector Borne Disease Control Programme (NVBDCP) recommended and field-tested case management and vector control strategies to eliminate malaria were used with some modifications based on local experiences and context. The detailed study design is presented in the companion paper [5].

### Development of the operational plan

The plan for MEDP was developed keeping the district as the operational and administrative unit. In the proposed model, the guidelines of the national programme [3] were followed to create a district-level operational plan. It includes the staffing, supervision, supply chain control and management and technical aspects of the elimination plan. In the existing system at district-level, the staff hierarchy starts from Accredited Social Health Activist (ASHA) at the village level, supervised by ASHA *sahayogi*, which is a helping-hand for the Auxiliary Nurse Midwife (ANM) at sub-center level that works with the Multipurpose Workers (MPW). There is one ASHA post sanctioned for every village. The ASHAs have a close-knit supervisory system in the form of ASHA-*sahayogis*, who monitor up-to five ASHAs. The ANMs and MPWs are active agencies in malaria surveillance.

**Fig. 2:**
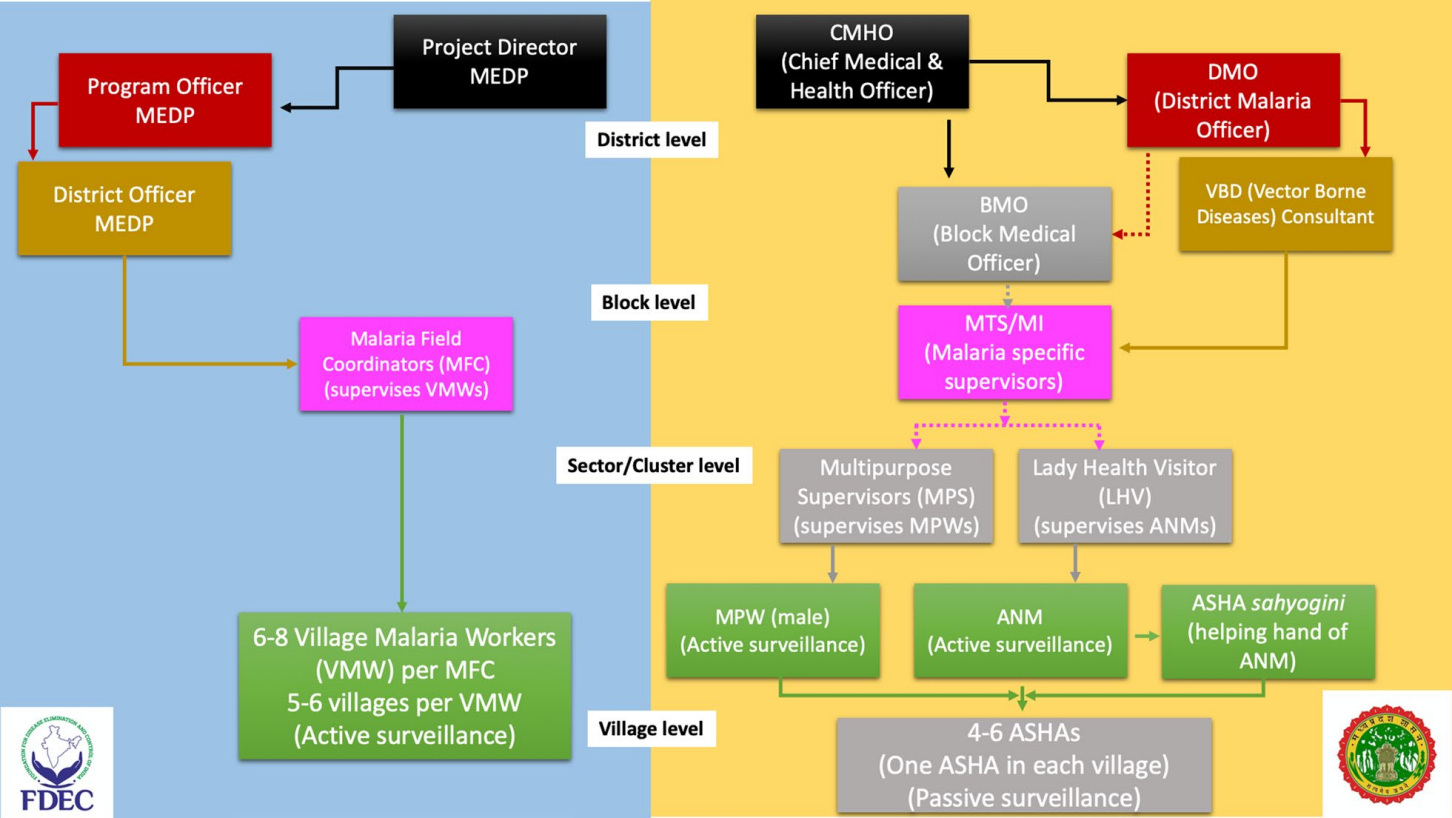
Comparative schematic of the malaria surveillance and case reporting system between the state government (right) and FDEC India (left) at district-level. Same color boxes indicate equivalent positions between the two systems

Both ANM and MPW report to the sector supervisors, which are Lady Health Visitor (LHV) and Multipurpose Supervisors (MPS). These workers report to the block-level Malaria Inspectors (MI) and Malaria Technical Supervisors (MTS), who finally report to the Block Medical Officer (BMO). The BMO corresponds with the District Malaria Officer (DMO) and the Chief Medical & Health Officer (CMHO) at the district-level. Roles and responsibilities of all government health functionaries working towards malaria elimination at district-level are summarized in Table 1. The DMO reports to the State Program Officer (SPO), which is a state level position. The SPO supervises the work of DMOs and reports to the Health Commissioner at the State level and to NVBDCP nodal officer at the central level. Figure 2 shows the malaria surveillance and case reporting system of state government at district level along with its MEDP counterparts.

**Table 1:** Roles and responsibilities of district-level government health functionaries working towards malaria elimination

Position	Roles and responsibilities
Chief Medical and Health Officer	Head of the district health administration. She/He is responsible for achieving the health goals in the district through appropriate planning, effective implementation and monitoring of all preventive and curative health care activities in the district.
District Malaria Officer	The key technical officer to handle the planning and monitoring of malaria elimination programme in the district. Reports to the Chief Medical and Health Officer.
District Vector Borne Disease Consultant	A technical officer from the National Vector Borne Disease Control Programme to assist the District Malaria Officer in discharge of his duties.
Malaria Technical Supervisors and Malaria Inspectors	Review of records in laboratory and malaria reporting forms (M Forms), review of vector control measures like IRS, bednets, Larvivorous fishes etc. Monitor logistics and utilization of drugs and commodities and report stock-outs. Reports to Block Medical Officer and District Malaria Officer,.
Multi-Purpose Supervisors and Lady Health Visitors	Monitoring and supervision of work-done by ANMs and MPWs. Reports to different block-level cadres for different health programmes.
Auxiliary Nurse Midwife (ANM) and Multi-Purpose Workers (MPWs)	ANMs and MPWs come with a broad set of responsibilities, including the support of AWWs (Anganwadi Workers) and ASHA workers. ANMs are primarily involved in Maternal and Child Health programmes. Both ANMs and MPWs are active surveillance agencies and supposed to regularly visit the villages on a periodic basis (door-to-door) and coordinate with the local ASHA towards different health needs of the community.
Accredited Social Health Activist (ASHA)	ASHA acts as a bridge between the ANM and the village and be accountable to the Panchayat. She is an honorary volunteer, receiving performance-based compensation for promoting universal immunisation, referral and escort services for RCH, testing and treatment of malaria, construction of household toilets, and other healthcare delivery programmes.
ASHA-*sahayogini* (helper)	Helps in the convergence of Integrated Child Development Services (ICDS) programme through the *Anganwadi* centres and the health department.

### Surveillance, reporting, monitoring, and feedback models

**Fig. 3:**
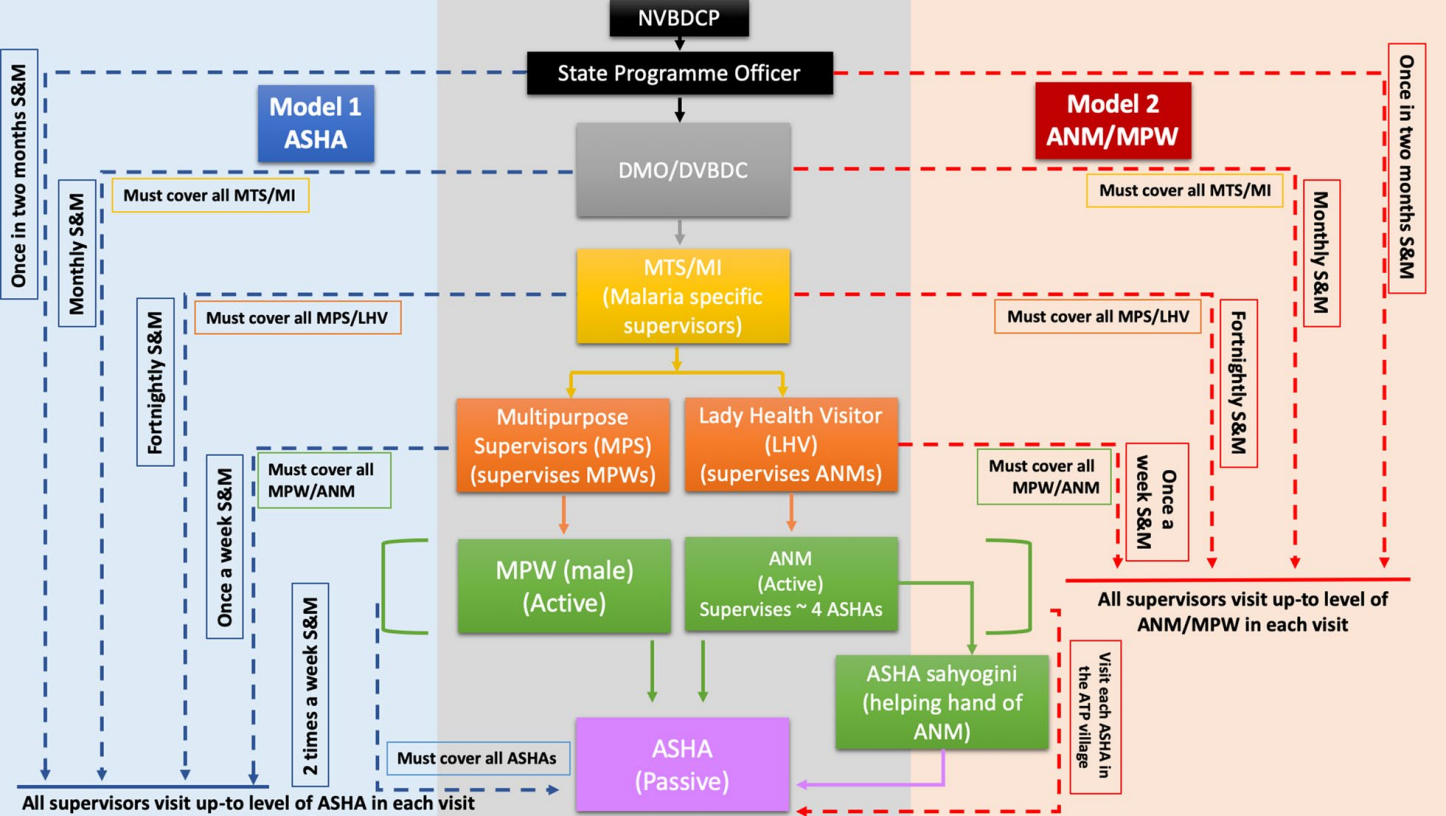
Two different model showing ANM/MPW and ASHA as the primary unit of surveillance

**Fig. 4:**
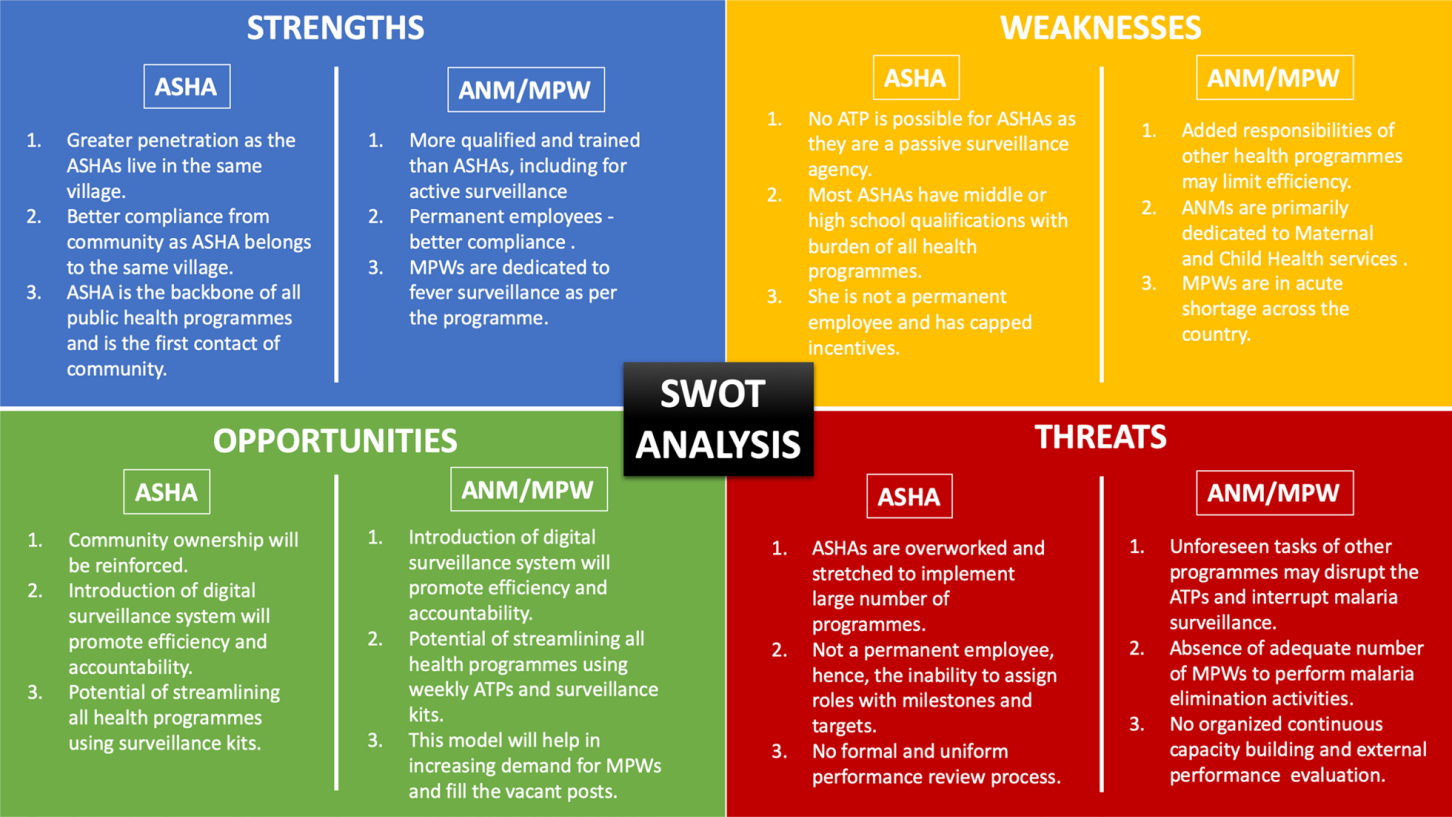
SWOT categorization of both ANM/MPW and ASHA-based models

Based on the learnings from MEDP, there are two models which can be used. One model revolves around the ANM/MPW as the first contact and the other model has the ASHA as the first contact. The plan ensures movement of the first contacts (ANM/MPW/ASHA) as per their Advance Tour Plans (ATPs) in the field. In the ANM/MPW model, one LHV/MPS will undertake responsibility of six ANM/MPW. These supervisors should visit two ANM/MPW each day. Whereas, in the ASHA model, the existing supervisory system of one ANM at subcentre looking over 6–8 villages/ASHAs should be used. Assuming a five-day work-week, supervisory visits will take three days, leaving two days for additional chores like trainings, reporting, meetings etc. A schematic of the models is described in Fig. 3 with the SWOT analysis of different models in Fig. 4. This supervision should: (1) check adherence to the ATP; (2) take feedback from the villagers about the performance and visits of the ANM/MPW; (3) initiate immediate disciplinary action in cases of unwarranted absence, disobedience, or poor work product; (4) check the status of supplies; (5) on-spot random inspections of tests performed by the front-line worker ; (6) perform random checks of the quality and methods of samples collected; and (7) cross-check the line-lists entered for the day with the respective residents of the village. During supervisory visits, all the grades should monitor up-to the level of the ANM/MPW and supervise the interim grades in-between the process. A sample of ATP of a VMW and MFC of MEDP along with district-level officials can be accessed on the project website [6].

### Implementation of a digital platform

MEDP developed a mobile surveillance tool by the name of SOCH–Solutions for Community Health workers. This application enabled the project to digitize all paper-based reporting systems, as well as enabled real-time data gathering and analysis, control on supply chain from base office to village worker and monitoring of staff [5].

### Diagnosis and case management

MEDP used the drugs approved by the National Drug Policy [7] and also subjected the RDTs to regular quality checks. The ground-level health staff were trained and equipped with the necessary modalities, A comprehensive field kit consisted of a bag pack containing all diagnostics and treatment supplies, uniform, umbrellas, IEC material, storage boxes, puncture-proof containers for storage of used lancets etc. More details about this kit have been described in a companion paper and is also available on the project website [5, 8].

In many areas, migration remained a challenge for malaria elimination. Each field worker during the visit to the respective household asked about the members living in the household. If anyone was out-of-station viz. outside their village or town, an active list of migratory population was maintained at the level of the ground-worker. This list contained the names and time duration of the people who have gone out for personal work or other reasons. The list also had the expected date of arrival for these populations and were immediately tracked upon their return [9].

**Fig. 5:**
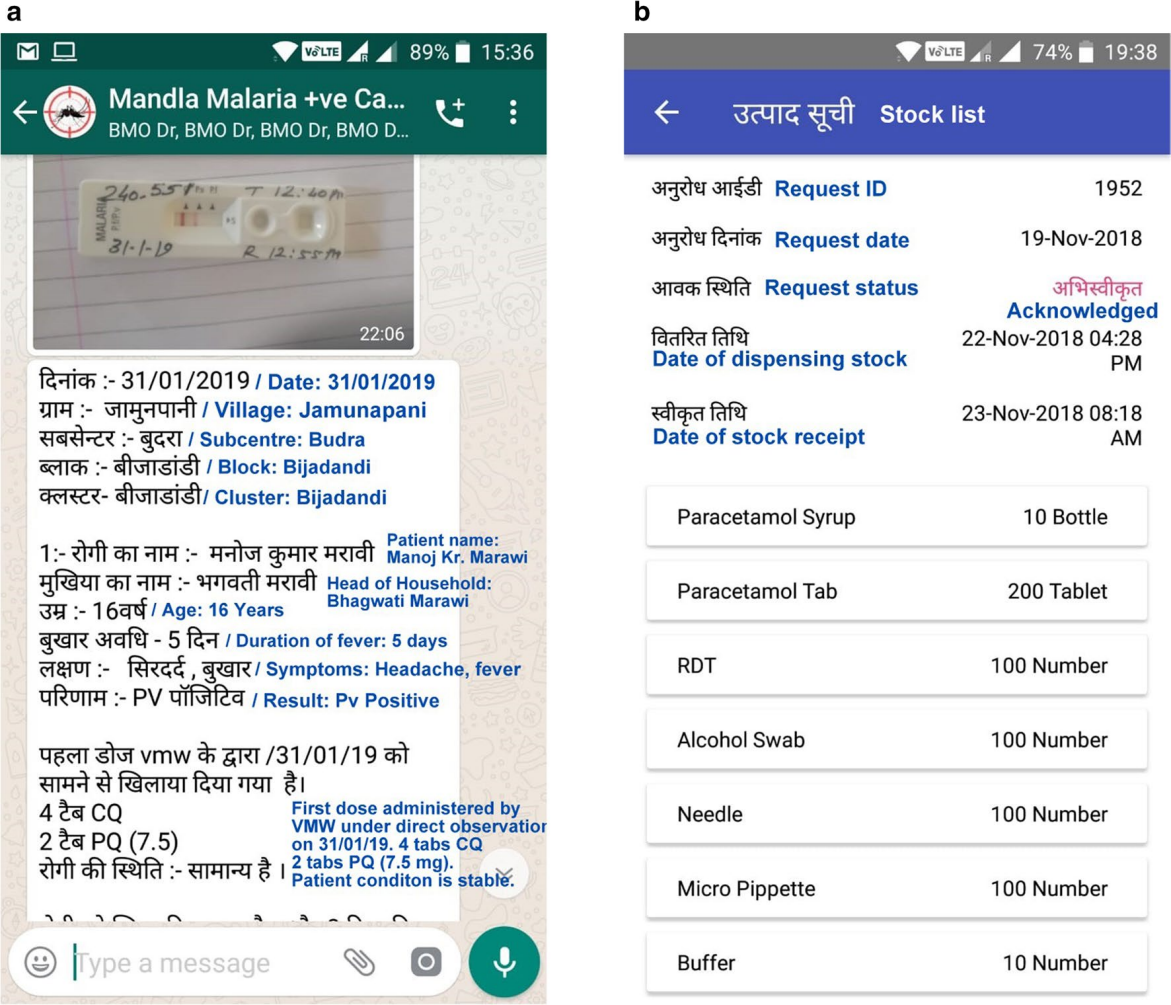
Real time cases information group in local language. English translation provided in blue colour

For communication, a WhatsApp group was created consisting of all personnel from SPO to the supervisory staff at district level. As soon a case was diagnosed, the information along with photo evidence was uploaded. The group was strictly dedicated to only malaria positive cases information and their follow-up. This group acted as a real-time information center for rapid action and response against malaria in the district. Sample screenshot is shared as Fig. 5a.

### Entomological aspect for vector control including monitoring of IRS and LLINs

The state mainly used Indoor Residual Sprays (IRS) and Long-Lasting Insecticidal Nets (LLINs) as the vector control measures. Emphasis was placed on educating communities on the benefits of minor engineering to reduce or eliminate breeding sites outside household. For the Indoor Residual Spray, the selection of the villages was done as per the API based on the guidelines laid down by NVBDCP. It should be kept in mind that NVBDCP recommended 45 days as the maximum duration for one round of spray. As per the learnings, it is recommended that the target should be 30 days with sufficient teams. This will ensure that early completion of the round 1 which will enable the programme to start the round 2 in time for the transmission season. Monitoring of the spray teams was done by the front-line workers with random inspections by all supervisory grades. LLIN distribution and post-distribution monitoring was performed by the front-line or its immediate supervisory staff. Random inspections by sector-level and above supervisory grades was also done.

### IEC that targets communities, schools, and local leaders

Each district has an IEC/BCC officer who is stationed in the office of CMHO at the district headquarters. He/she is the nodal person for IEC/BCC malaria activities in the district for state health authorities. Some of the groups which were targeted included: (1) School teachers as influencers for children; (2) Middle school students (standards six to eight) as children below sixth standard may be too young to comprehend the messages and a children above eight standard have significant academic pressure to entertain IEC/BCC campaigns and contribute in the same; and (3) Community through weekly community markets using portable IEC/T4 (Track fever, Test fever, Treat patient, Track patient) booths and interpersonal communication during house-to-house visits [5, 10].

### Training

MEDP conducted four to five-day induction trainings for new hires and yearly refresher trainings for existing field staff. The minimum passing threshold for post-training assessment was 70 %. Each participant performed at-least five RDTs, blood slide collections, and filter paper collections each. No batch was for more than 40 candidates. These trainings were assessed using a pre and post-test questionnaire with minimum passing threshold to determine impact of training and materials for subsequent training. Since all village level workers had mobile cell phones, regular messages about malaria surveillance, testing and reporting were made part of the training [10] Assessment papers were checked on the same day of the exam and failed candidates were re-trained the next day. The candidate was cleared for duty only when she/he had passed the test. If she/he failed after two attempts, the candidate was let go, and a new person was recruited for the position. Additionally, refreshers before any major vector control exercise such as distribution of LLINs and IRS were done. Detailed training schedule and pre/post training assessment questionnaires are available on the website [11, 12].

### Supply chain management

While paper-based systems for requisitions and management already exists; the SOCH digital system or a comparable system, is recommended. This system added excellent management and HR controls to the existing system. The mobile application used by the front-line worker (ASHA/ANM/MPW) can enable them to use the app for requesting/acknowledging and tracking their stock consumption. The request can be raised by the worker and it escalates up-to the supply chain in-charge at the district level after interim supervisory approvals. Figure 5b shows a sample for request raised by a front-line worker. This helps in tracking the stock status in real time throughout the field personnel. In order to ensure uninterrupted supply to the front-line workers, it is a good practice to make the supervisory grades a depot for buffer stock that may be needed by their staff.

**Fig. 6:**
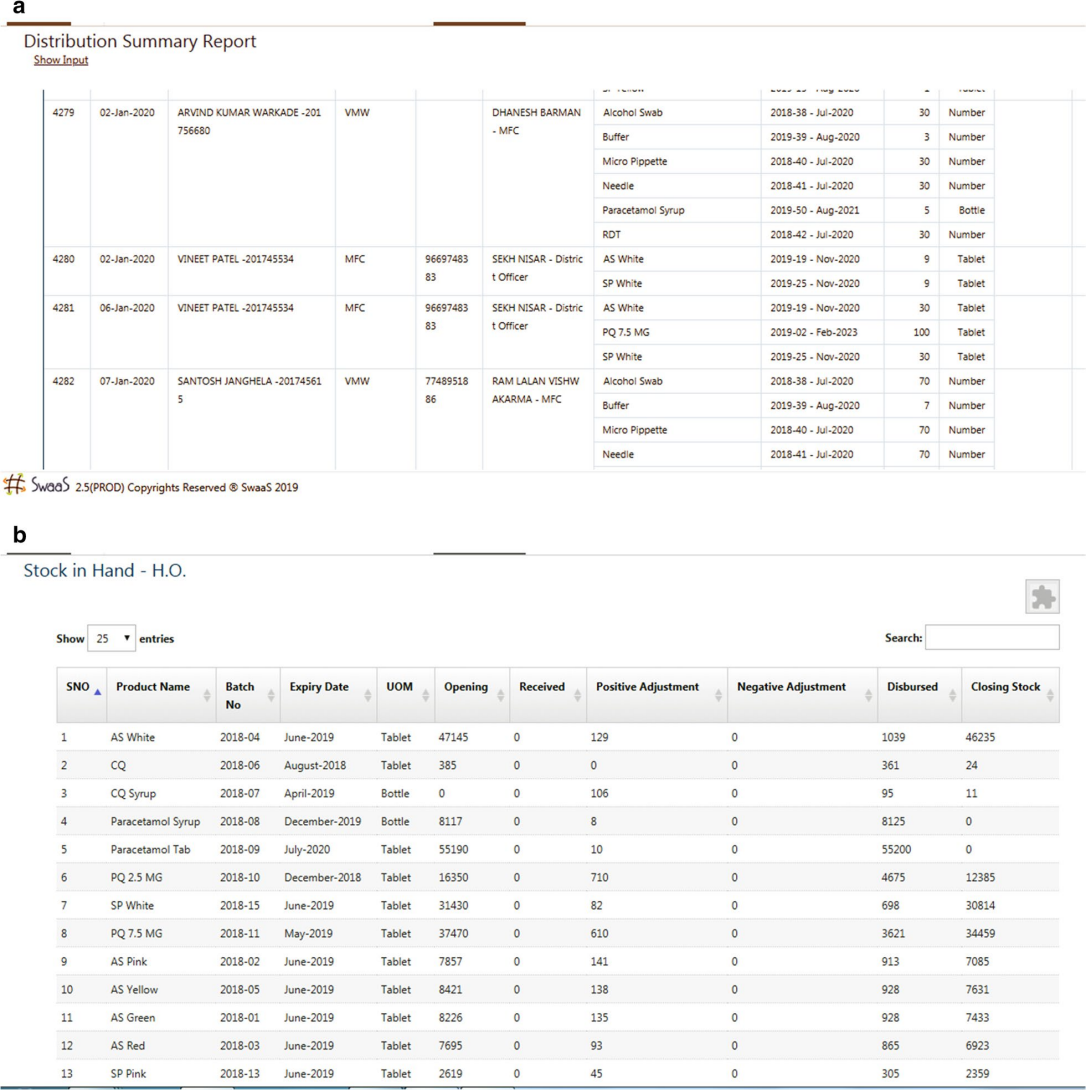
Hardcopy data reporting model at district-level

In a month, based upon average usage, all stock requisitions were requested on any two days. This prevented flooding of requests and helped in keeping track of requests from the entire district. When all the requests came on 24/25 of the month, the stock was prepared for distribution during the monthly meeting of supervisors on 2nd of every month. Figure 6a is a snapshot of report generated for stock distribution vouchers. Figure 6b is a snapshot of report generated for stock at the district office. At MEDP, it was found that having real-time information of stock-at-hand to the last level of field staff helps in maintaining the crucial supply chain and ensure non-interruption of services owing to stock problems. The system also allows robust accountability at every level.

### Review process on weekly and monthly basis against set milestones

 Regular reviews were a part of good monitoring and evaluation practices. Continuous reviews helped in identification of practices yielding desired results and course correction, if required. Table 2 summarizes a proposed review plan across all levels of the programme. Documentation of data and submission of reports was done on weekly and monthly basis. MEDP endorses the use of mobile application surveillance tool because of its in-built data validation functionalities which ensured optimal and real-time data. The major challenge in using hardcopies was timely delivery of the recorded data to the appropriate authorities. This included collection of reports from all ground-level units, delivery, digitization and validation of the data [13].

**Table 2:** Proposed schedule of continuous reviews for the malaria programme

S.No.	Project component/process	Frequency	Mode	Follow-up/action taken/review
1.	Overall project progress – National/State level review	Annual	In-person	In subsequent months
2.	Supervisory-level monthly meeting	Monthly	In-person	Immediate
3.	30-point monitoring checklist	Monthly compilation	Hardcopies – digitized within same month	In subsequent months
4.	Monthly project progress reports – includes surveillance data and major highlights of the month	Monthly	Electronic	In the subsequent month.
5.	ANM/MPW meeting at cluster-level	Fortnightly	In-person	Immediate
6.	Weekly project progress report – includes work-done, work-planned, open action items and detailed field visit reports	Weekly	Electronic report followed by in-person conference every Monday	Immediate
7.	Findings during field visits	Weekly	Electronic reports and in-person discussions	Immediate
8.	Work report of each MPS/LHV and MPW/ANM with highlights	Daily	Electronic on SOCH app and WhatsApp	Immediate
9.	Monitoring and feedback of work-products through mobile app and WhatsApp	24 × 7	Verbal and electronic through WhatsApp	Immediate

The following mechanism for efficient collection of the data at the district-level is proposed. All sector supervisors should meet the concerned ANMs/MPWs at sector-level every Saturday to collect the hardcopy data. Following this, a block-level meeting should be held on Monday. The data should be then submitted to the MTS/MI, who should then submit it to the BMO, who should send it to the DMO/DVBDC. The reports must go on a monthly basis to the SPO and NVBDCP nodal officer for the respective state.

No data re-conciliation requests should be entered as a routine process at the end of the month or end of the year. This will ensure a level of seriousness and sincerity in reporting the data within the stipulated time-frame at every level. On Saturday, MPS/LHV should collect all the hardcopies and segregate them based on time period and location, and next day (Monday), these materials should be handed over to the MTS/MI, who must get this data counter-signed by the BMO along with the data from the CHC laboratories. On Tuesday, these data sets from blocks of the district, should be sent to the district HQs by the first bus in the morning. The bus details should be communicated to the clerical staff at the DMO office. After receiving the data, clerical staff should communicate to the respective MTS/MI.

**Fig. 7:**
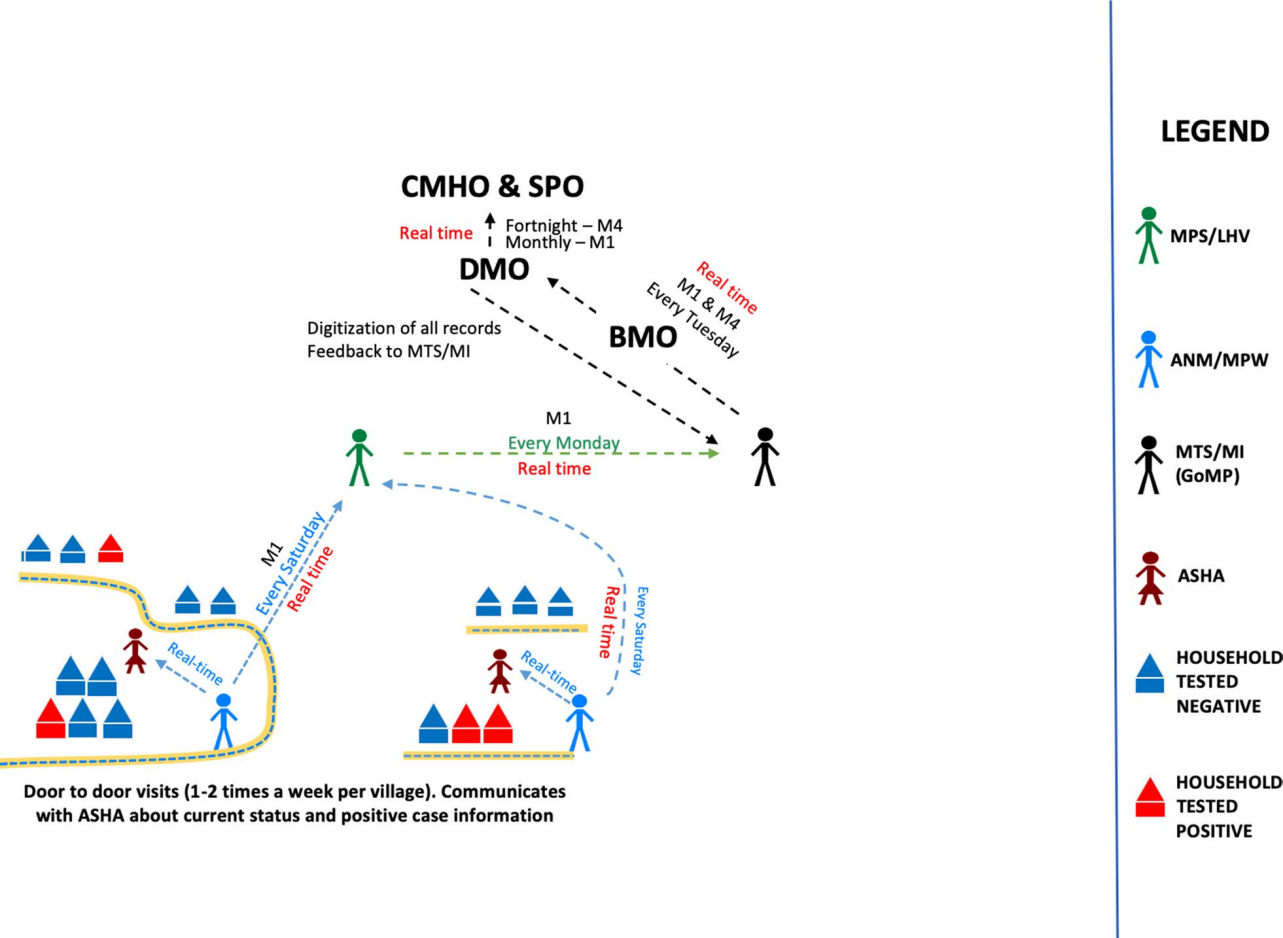
Stock request from front line worker through the mobile app. English translation written adjacent to local language text

In this manner, the disease surveillance and treatment data will be sent every Tuesday to the district HQs—three times a month and the fourth week data will be hand-delivered by the MTS/MI during the monthly meeting at the DMO office. The monthly meeting is a good opportunity to present the findings from the past three weeks of data submitted by the MTS/MI. Any irregularities in the data must be reported and discussed in this meeting. Figure 7 shows a proposed model for hardcopy data reporting. It should be noted that all positive cases information must be communicated in real-time. This can be through a telephone call from ASHA/MPW/ANM to the MPS/LHV/MI/MTS, who should take the lead and report it in-writing on a WhatsApp group or Email with copy to all concerned personnel.

### Independent review of program and monitoring progress

MEDP hosted an independent review each year in the form of a Malaria Elimination Advisory Group meeting [5]. This was a two to three days meeting which critically reviewed each aspect of the project and provided its recommendations. In the proposed model, similar reviews can be organized at intra-government level, where other districts and states review the progress of their counterparts. These can also be third party scientific or private sector institutions.

## Results

MEDP has developed a detailed and tested operational plan describing each component of its malaria elimination goal. It describes the strategies viz. surveillance and case management, vector control, capacity building, IEC/BCC, SOCH mobile application, and monitoring and evaluation [5]. Between June 2017 to May 2020, MEDP has reported a reduction of 91 % indigenous malaria cases in Mandla. Several rounds of mass screenings were conducted and revealed 0.18 % positivity in Sep-Oct 2018, followed by 0.06 % in June 2019, and 0.03 % in December 2019, and these were mostly asymptomatic cases in the community. The district has a total of 297 sub-centres, out of which, in the year 2017, 143 sub-centres were free of malaria, which increased to 198 in 2018, and 211 in 2019 [9].

Periodic entomological investigation revealed several key findings such as prevalence of the vector, effect on seasonal variations on vector density, status of insecticide resistance in the district, and malaria positivity in the anopheline population [14]. Significant improvement in the mosquito knockdown rates and usage of LLINs was observed following monitoring of vector control measures (IRS and LLIN) by MEDP.

The project has trained over 422 field staff and achieved qualifying rate of 94.3 % after a single training session. Significant and progressive improvement in scores of existing and new staff has been noted with techniques such as ‘shadowing’ and introduction of new monitoring tools (30-point checklist) [10]. Using the SOCH mobile application, the project enrolled the entire population of the district (1.15 million) and assigned UIDs to each individual [15]. Using this tool, the project undertook a detailed study on the socio-economic factors affecting malaria and found association between malaria cases and different household variables, such as age, gender, number of members, number of rooms, caste, type of house, toilet facilities, water supply, cattle sheds, agricultural land, income, and vector control interventions [15].

The mobile application also improved the stock accountability by 60 % and adherence to the Advance Tour Plans from 62–95 %. After the roll-out of the application, the weaning off from paper-based reporting systems took nine months. During this time, the initial difference in reporting between paper-based system and mobile-app systems was 49.6 %, which was reduced to 0.4 % with regular troubleshooting. Detailed description and results of the SOCH system have been discussed in companion paper belonging to the same thematic series.

MEDP also conducted an ASHA needs-assessment and found serious lacunae in malaria diagnosis and treatment practices of ASHA workers of Mandla district. Only 15 % and 10.5 % ASHAs correctly identified malaria parasites as *Plasmodium vivax* and *Plasmodium falciparum* ‘positive’, respectively. The study also showed that 19.1 % ASHAs did not have any RDTs with them and 47.7 % did not have any ACT for treatment of *P. falciparum* cases, which is the dominant malaria infection in the district. Following this needs-assessment, MEDP created a comprehensive training module for ASHAs. The module attempts to address all the gaps identified during the study. Both the needs-assessment study and training module have been submitted for publication as part of the MEDP thematic series.

## Discussion

The key objective of this paper was to propose a replicable model for malaria elimination, based upon the learnings of the Malaria Elimination Demonstration Project, Mandla, Madhya Pradesh. This demonstration project achieved more than 90 % reduction in indigenous malaria cases within a short span of time.

Two surveillance models have been proposed – one with the ANM/MPW as the first contact and the other model proposed ASHA worker as the first contact. In both models, it may be argued that added responsibilities from other national health programmes may limit their efficiency. The total staff strength in Mandla is 2,184, as part of the state government that works full-time or part-time on malaria control as compared to the MEDP staff strength of 260 field staff. If the staff strength of other malaria affected districts is similar, additional staff may not be required.

The key need is to have work responsibility and accountability from staff at all levels for the stated goals of the elimination project in a manner that meets milestones and targets. Based upon the learnings of MEDP, if the staff has a fixed Advance Tour Plan (ATP) and the complete kit with them, they can cater to all the responsibilities in a single visit.

The Advance Tour Plans (ATPs), which are pre-determined plans for movement of the field staff from ANMs to the DMO should be clearly established and used for supervision of staff. It was observed at MEDP that a strong adherence to the ATPs can: (1) help development of a strong rapport with the community; (2) help tracking of field staff by supervisors in areas with poor mobile phone network; and (3) instill discipline and accountability in the field staff. Supervisor ATPs (LHV/MPS and above) should be reshuffled every month and the updated ATPs should be shared up-to the top-level.

A comprehensive monitoring checklist is also recommended, which should be filled by the supervisor during every visit to their field staff. A sample of such a checklist can be seen on the project website [16].

The following lessons from MEDP would be helpful: (1) Ensuring that the field staff is fully equipped with all the commodities ensures no gaps during delivery of their duties on a regular basis. In the needs-assessment study of the ASHAs, it was noted that several ASHAs did not have adequate stock of anti-malarials and diagnostics which led them either to dispense insufficient/improper treatment or did not test the patient altogether; (2) The special field exercises such as mass screenings should provide total supervision and monitoring support to the teams. It has been seen at MEDP, once the district-level officials are leading the teams on the field and are involved in T4 and advocacy efforts with the field staff, the results are improved with much better enthusiasm, morale, and compliance at every level; and (3) MSAT can also be used in hard-to-reach areas, which are inaccessible during floods or due to hard terrains, as preferred mode of intervention. This can be done instead of regular surveillance. The MSAT can be planned up-to three times in a year (pre-transmission, during transmission and post-transmission) to resolve any reservoirs of infection.

At MEDP, during the Indoor Residual Sprays, it was observed that a spray plan with no buffer degraded the spray quality as the teams looked forward to only completing the targets. The plan should be created with buffer for various field-challenges like rains, travel time from one block to another, operational difficulties, troubleshooting of equipment, and non-cooperation from community. Additionally, a mop-round to cover the missed houses during first attempt should be inserted in the plan. MEDP also used a checklist for monitoring the spray operations and the same can be accessed on the project’s website [17].

For the distribution and monitoring of the LLINs, the key recommendation is to monitor the post-distribution usage and perform regular IEC/BCC activities focusing on the same. Planning for distribution should be done at-least 15 days before arrival of the LLINs. Sufficient number of nets to cover each household member should be provided. At the day of the distribution, a minimum of one supervisor of the respective sector/sub-center/block must be present at the time of the distribution. A team of field staff should be designated for opening the packet, briefing the usage/storage/washing methods to each and every user. No LLIN must be distributed without opening the packets. Immediately after distribution, the staff visiting the village as per the ATP should start IEC/BCC activities for proper usage of the bed nets. The residents should be asked to take out the bed-nets, the method of hanging them should be demonstrated and strict advice for using the same must be given. At every six months interval, the supervisory staff should conduct a post-distribution usage monitoring survey in a representative sample of the population. This survey will include physically verification of bed-nets for its distribution, condition, availability, usage and washing pattern. To assess the actual usage of LLINs, provisions of hanging the bed-nets should be checked. A sample monitoring checklist for LLINs is provided on the project website [18].

Quarterly entomological monitoring of the area is also recommended. This should involve resting and night collections, insecticide resistance status, and vector incrimination [14]. Findings from these studies will be essential to know the resting and feeding behaviour of local vectors and insecticide resistance status. These findings will be helpful during preparation of the national/state operational plans for malaria elimination. It was observed at MEDP that IRS quality and mosquito knock-down rate improved significantly after supportive supervision by the project.

The IEC/BCC and capacity building is significant and if done properly in a community-context manner, it will prepare communities and community leaders to seek health care. In the context of malaria elimination, once the programme enters into elimination or post-elimination phases, funding is likely to go down and the efforts may dilute. That is the time when the investments made in IEC/BCC of the community will provide the best return on investments. The tools should be developed on the principle of ‘*for the people, by the people’*, implying that the messages should be drafted after taking feedback from the community during initial surveys. The few tools that have been developed by MEDP, are free to use by any stakeholder and the project does not retain any copyrights on these tools [19].

Following the roll-out of these activities, pan-district yearly assessments should be conducted with study groups consisting of samples from the target population. This should be followed by re-orientation and course-correction trainings. A sample of the IEC/BCC KAP assessment tool is provided on the project website [20].

MEDP has also offered the SOCH mobile application free-of-charge to the state government of Madhya Pradesh and asked the NVBDCP to evaluate the same for pan-India implementation. A schematic for seamless integration of SOCH into the existing health systems has also been shared with the state government for development of a One Data One Reporting (ODOR) system.

The following lessons from MEDP would be helpful in developing training materials for the field staff: (1) A needs-assessment study conducted by MEDP for the ASHAs of Mandla district revealed that only 55 % ASHAs had received malaria trainings. Only 10 and 15 % ASHAs could identify the *Plasmodium vivax* and *Plasmodium falciparum* RDTs correctly. 60–65 % ASHAs could not tell the correct colour of ACT packs for different age groups; (2) A robust pre/post-test questionnaire with high qualifying threshold ensures quality in the trainings imparted and also acts as an indicator of the trainee as well as the trainer’s performance [10]; (3) MEDP regularly used the above-mentioned methods in its trainings to the Village Malaria Workers and witnessed a 95 % qualifying rate keeping a 70 % passing threshold in pre/post-tests [10]; and (4) It was also noticed that the field staff forgot the malaria dosages faster than any other operational component. This was due to the technical nature of information and due to absence of cases in his/her field area. Regular revisions during spot field visits and refresher trainings are recommended. A training module for the ASHAs has already been developed by the project and will be published in companion papers.

Malaria drugs approved by the National Drug Policy only should be used [7]. It has been observed that AYUSH anti-malaria drugs are being used in some places, which have not gone through appropriate pre-clinical, toxicity, safety and efficacy studies and are not approved by the national drug regulator. These drugs pose credible threat to malaria elimination efforts as their effectivity is unproven and spread distrust in the community. Additionally, regular quality control of each batch of diagnostic tools (RDTs) used by the programme is essential.

The following lessons learned from the MEDP may be considered: (1) MEDP follows a strict schedule of continuous reviews. The project has not missed even a single weekly review since the inception of field operations in September 2017. This has helped in achieving the goals and objectives in a time bound manner. These review meetings are chaired by the Project Director, irrespective where he is in India or outside India, demonstrating the importance of review; (2) The mobile application SOCH provides 100 % data accountability and robustness in the system. MEDP has phased out all paper-based reporting systems; and (3) MEDP also maintains a strict daily reporting of the field staff. Apart from the data reported through SOCH, the field supervisors are supposed to deliver a short report of the day’s activity in a WhatsApp group consisting of the District officials. Similarly, all ground-level staff (VMWs) are supposed to deliver a similar report in their respective cluster group which is monitored by the respective supervisor and the District Officer.

The global malaria elimination community is fortunate to have highly effective diagnostic tests that detect malaria parasites (*P. falciparum* and *P. vivax*) and combination drug regimens that are highly effective in treatment and prevention of malaria. India makes these diagnostic tests and drugs, which should make elimination program an accomplishable goal from supply chain perspectives. India also has recent experiences of successful polio elimination and HIV/AIDS control, which are applicable from operational and management considerations [21–23]. The need for prioritizing and addressing the research gaps and work closely with the national program has been well identified and addressed by the Malaria Elimination Research Alliance India (MERA India) [24]. The emerging challenges for malaria elimination are well known and need to be discussed and inserted into the strategic plans at national level. Capacity building of state/district level officials to successfully execute and monitor these plans is equally important [25].

There are several challenges which may act as barriers in successful replication of the proposed models for malaria elimination. These include: (1) Motivating the staff to switch to digital reporting from paper-based reporting systems. This was faced at MEDP during the SOCH application roll-out. This barrier was resolved with continuous monitoring, motivation, and troubleshooting; (2) Confluence of multiple national programmes at the level of front-line health staff. This is the biggest challenge and will require a strategy to integrate the objectives of the health programmes into the ATPs of the front-line health staff; (3) Continuous monitoring, evaluation, learning, and feedback is required at every level. MEDP has prepared a Monitoring and Evaluation (M&E) framework, which can be accessed from Additional file 1: Annexure 1; (4) Supply chain management will enable the front-line health staff to discharge their duties efficiently. Although the digital stock management system will help, but any lapses in the supply chain should be reviewed seriously from the M&E standpoint; and (5) Lack of quality training in the current system. The best way to address this lacuna is to link the performance of the trainees in the qualifying examinations with the trainer’s skills and efforts.

## Conclusions

Malaria elimination is a high-priority public health goal of the Indian Government, with commitment for elimination by year 2030. The elimination of malaria by this date, or even earlier, is an accomplishable goal. But, if the national programme is not appropriately resourced with accountability, there is a risk of missing the 2030 target [26].

As the elimination goals advance at the state and central government levels, the following emerge as the important aspects for consideration at programmatic, policy and political levels: (1) There is need for a model derived from real field experiences towards malaria elimination; (2) The programme has adequate resources at state and local levels to put into practice the elimination programme; (3) The model may be based on two ways–keeping ASHA as front-line worker or keeping ANM/MPW as front-line workers; (4) Unlearning of wrongful psychological and physiological practices is needed before fresh trainings can be initiated; (5) Trainings must include a pre and post assessment with at least 70 % qualifying threshold; (6) Entire staff from DMO to the ASHA/ANM/MPW must adhere to a strict Advance Tour Plan; (7) Digital data reporting can be adopted in the system using a mobile application surveillance tool already in place. Efforts are needed to improve data quality and reliability; (8) IEC/BCC campaigns should be designed as per the requirements of the community; (9) Strict monitoring and supervision of implementation of vector control measures such as IRS and LLIN is required; (10) Proper equipment and availability of supplies at field level is necessary for efficient case management and treatment; 11) Mass screenings should be undertaken at least thrice a year in high API and hard-to-reach sub centres; 12) Digital supply chain management can help in improving the reliability and data accuracy of the system; 13) Regular and periodic reviews can help in improving overall work product. These start from daily to annual reviews; 14) Ownership of the programme is required from the ground level up-to the level of PS Health; and 15) The elimination programme would depend on effective management, operational, technical and financial controls, so that staff at all levels are held accountable for the work elements and milestones for which they are responsible.

## Supplementary Information

**Additional file 1.** Key performance indicators (KPIs) for malaria elimination.

## Data Availability

We have reported all the findings in this manuscript. The hardcopy data is stored at MEDP Office in Mandla, Madhya Pradesh and Indian Council of Medical Research - National Institute of Research in Tribal Health (ICMR - NIRTH), Jabalpur, Madhya Pradesh. Softcopy data is available on the project server of MEDP hosted by Microsoft Azure. If anyone wants to review or use the data, they should contact:

## Abbreviations

ANM: Auxiliary Nurse Midwife
ASHA: Accredited Social Health Activist
ATP: Advance Tour Plan
BMO: Block Medical Officer
CMHO: Chief Medical & Health Officer
DMO: District Malaria Officer
FDEC: Foundation for Disease Elimination and Control of India
ICMR: Indian Council of Medical Research
IRS: Indoor Residual Spray
LHV: Lady Health Visitor
LLIN: Long-Lasting Insecticidal Net
MEDP: Malaria Elimination Demonstration Project
MERA India: Malaria Elimination Research Alliance India
MI: Malaria Inspector
MPS: Multi-Purpose Supervisor
MPW: Multi-Purpose Worker
MTS: Malaria Technical Supervisor
SPO: State Programme Officer
