# Supplementary material for: A model for malaria elimination based on learnings from the Malaria Elimination Demonstration Project, Mandla district, Madhya Pradesh
Source: Malar J. 2021 Feb 16;20:98. doi: 10.1186/s12936-021-03607-3 (PMC7888092; doi:10.1186/s12936-021-03607-3)
Supplement: Supplementary file 1 — Additional file 1. Key performance indicators (KPIs) for malaria elimination. [file 12936_2021_3607_MOESM1_ESM.docx]

**ANNEXURE 1: KEY PERFORMANCE INDICATORS (KPIs) FOR MALARIA ELIMINATION**

KEY PERFORMANCE INDICATORS:

The Performance Monitoring Plan (PMP) presented in the table below provides indicators (operational plan and others) that are useful for tracking and managing program activities and their immediate and intermediate results. We also adopted impact indicators from Global Technical Strategy (GTS) and National Framework for Malaria Elimination (NFME).

**Key area 1:** Surveillance and Case Management (Detection and Treatment)

| **Level** | **Indicator** | **Definition** | **Data Source** | **Target** | **Frequency** |
| --- | --- | --- | --- | --- | --- |
| **Inputs** | Proportion of funds available as against sanctioned  *(to be segregated for each activity under surveillance & case management)* | Funds available | Project |  | Monthly |
|  |  | Funds sanctioned |  |  |  |
|  | Proportion of health personnel in post as against sanctioned  *(to be segregated for every staff category under surveillance and case management)* | Number of health personnel in post | Project |  | Quarterly |
|  |  | Number of health personnel sanctioned |  |  |  |
|  | Proportion of Rapid Diagnostic Tests (RDTs) made available to project | Total RDTs received by the programme in the district | Logistic Information System | Need based | Annually |
|  |  | Total RDTs planned for procurement |  |  |  |
|  | Proportion of anti-malarial drugs made available to project | Total anti-malarial drugs received by the programme in the district | Logistic Information System | Need based | Annually |
|  |  | Total anti-malarial drugs planned for procurement |  |  |  |
| **Process** | Proportion of funds utilized as against available | Funds utilized | Project |  | Monthly |
|  |  | Funds available |  |  |  |
|  | Proportion of training programs conducted for Health personnel  *(diagnosis, treatment, surveillance, logistics management etc)* | Training sessions conducted | Training data base | Y 2- 80%  Y 3-100% | Quarterly |
|  |  | Training sessions planned |  |  |  |
|  | Proportion of RDTs supplied to Accredited Social Health Activist (ASHA)/ Auxiliary Nurse Midwife (ANM)/ Multi-Purpose Worker (MPW) as per requirement | Number of RDT supplied | Logistic Information System | Need based | Quarterly |
|  |  | Number of RDT required |  |  |  |
|  |  | Total number of ASHA/ ANM/MPW |  |  |  |
|  | Proportion of ASHA/ ANM/MPW supplied with indented number of anti-malarial drugs | Number of VMWs supplied with indented number of anti-malarial drugs | Logistic Information System | Y1(onwards)-100% | Quarterly |
|  |  | Total number of VMWs |  |  |  |
|  | Proportion of Sub-centers (SCs) with required stock of all anti-malarial drugs | Number of SCs with recommended number of anti-malarial drug | Periodic assessment form | Year 2- 60%  Year 3-100% | Quarterly |
|  |  | Total number of SCs |  |  |  |
|  |  | Total ASHA/ ANM/MPW monitored |  |  |  |
|  | Proportion of SCs with all supplies required for performing parasitological test | Total SCs with material for slide preparation/RDT | Periodic assessment form | Year 2- 60%  Year 3-100% | Quarterly |
|  |  | Total SCs monitored (only applicable for functional sub centers) |  |  |  |
|  | Proportion of Mass Screening and Treatment (MSAT) camps conducted as against planned | Number of MSAT camps conducted | Project |  |  |
|  |  | Number of MSAT camps planned |  |  |  |
| **Output indicators** | Fever cases among screened population | Total fever cases found | Active Case Detection (ACD) |  | Monthly |
|  |  | Total population screened |  |  |  |
|  | Trained health personnel  (This indicator will be segregated for ASHA and ANMs) | Total provider trained | Training data base | Y 2- 80%  Y 3-100% | Half yearly |
|  |  | Total providers targeted |  |  |  |
|  | Proportion of ACD conducted as per plan | Total ACD conducted | Monthly planner | 100% as targeted | Monthly |
|  |  | Total planned |  |  |  |
|  | Annual Blood Examination Rate (ABER) | Total blood examination in a year | ACD and PCD format | >10% | Yearly |
|  |  | Total population |  |  |  |
|  | Proportion of patients with suspected malaria who receive a parasitological test* | Total RDT done of fever cases | ACD | Year 2- 60%  Year 3-100% | Monthly |
|  |  | Total fever cases found during ACD |  |  |  |
|  | Proportion of asymptomatic malaria infection | Total malaria cases found using the RDT in asymptomatic population during MSAT | MSAT reporting format (ACD form will be used) |  | Quarterly |
|  |  | Total asymptomatic population subjected to RDT in during MSAT |  |  |  |
|  | Proportion of cases received malaria treatment | Total malaria cases received treatment | ACD and PCD format | Y1- 80%  Y2- 100% | Monthly |
|  |  | Total malaria positive cases |  |  |  |
| Outcome | Delay in reporting for treatment | Total malaria positive cases with onset of fever more than 3 days | ACD and PCD format | Year 2- 25%  Year 3 - 10%  Year 4 - <1% | Quarterly |
|  |  | Total malaria positive cases |  |  |  |
|  | Proportion of health personnel know proper use of RDTs  [Indicator will be segregated for ASHA, ANMs and MPW] | Health personnel knowing proper use of RDT | Periodic assessment form | Y1- 60%  Y2- 80%  Y3- 100% | Quarterly |
|  |  | Health personnel assessed |  |  |  |
|  | Proportion of health personnel know proper use of anti-malarial drugs  [Indicator will be segregated for ASHA, ANMs and MPW (and RMP)] | Health personnel knowing proper use of anti-malarial drugs | Periodic assessment form | Y1- 60%  Y2- 80%  Y3- 100% | Quarterly |
|  |  | Health personnel assessed |  |  |  |
|  | Proportion of malaria positive patients received Appropriate malaria treatment ** | Total malaria cases received appropriate treatment | ACD and PCD format | Y1- 80%  Y2- 100% | Monthly |
|  |  | Total malaria positive cases |  |  |  |
|  | Timeliness of report | Total reports received on time | Sentinel sites report | Y1- 60%  Y2- 80%  Y3- 100% | Monthly |
|  |  | Total reports received |  |  |  |
|  | Proportion of cases investigated within 7 days ***(elimination phase)*** | Number of cases investigated in elimination phase within 7 days |  |  |  |
|  |  | Total number of cases found |  |  |  |
|  | Proportion of foci investigated within 7 days ***(elimination phase)*** | Number of foci investigated in elimination phase within 7 days |  |  |  |
|  |  | Total number of foci found |  |  |  |
|  | Malaria case incidence**  (Annual Parasite Incidence) | Total Malaria positive cases in a year | ACD and PCD formats | Y2- 50%  Y3-60%  Y4-70%  Reduction to be calculated considering previous year as baseline | Annually |
|  |  | Total Population |  |  |  |
|  | Parasite-prevalence**  Proportion of children aged 6-59 months with malaria infection | Number of children aged 6-59 months with malaria infection detected by microscopy or RDT | ACD | Y2- 50%  Y3-60%  Y4-70%  Reduction to be calculated considering previous year as baseline | Annually |
|  |  | Number of children aged 6-59 months tested for malaria parasites by microscopy or RDT |  |  |  |
|  | Malaria positive pregnant women** | Total malaria positive in pregnant women | ACD and PCD formats | Y2- 50%  Y3-60%  Y4-70%  Reduction to be calculated considering previous year as baseline | Annually |
|  |  | Total pregnant women who were tested for malaria during Ante Natal Care (ANC) /ACD |  |  |  |
| **Impact** | Annual falciparum Incidence rate (*Pf*) | Total *P. falciparum* cases | ACD and PCD formats | Y2- 50%  Y3-60%  Y4-70%  Reduction to be calculated considering previous year as baseline | Annually |
|  |  | Total malaria cases |  |  |  |
|  | Incidence of severe disease # | Malaria cases with any severe complication admitted to sentinel sites | Sentinel surveillance | Y2- 50%  Y3-60%  Y4-70%  Reduction to be calculated considering previous year as baseline | Annually |
|  |  | Total malaria positive cases |  |  |  |
|  | Malaria mortality rate** (per 100,0000 population) | Deaths due to malaria |  | Y2- 50%  Y3-50%  Y4- 100%  Reduction to be calculated considering previous year as baseline | Annually |
|  |  | Total population |  |  |  |
|  |  | Total Malaria cases |  |  |  |

**Key area 2:** Prevention/ Control of Vector

| **Level** | **Indicator** | **Definition** | **Data Source** | **Target** | **Frequency** |
| --- | --- | --- | --- | --- | --- |
| **Input** | Proportion of funds available as against sanctioned  *(to be segregated for each activity under vector control)* | Funds available | Project |  | Monthly |
|  |  | Funds sanctioned |  |  |  |
|  | Proportion of health personnel in post as against sanctioned  *(to be segregated for every staff category under vector control)* | Number of health personnel posted | Project |  | Quarterly |
|  |  | Number of health personnel sanctioned |  |  |  |
|  | Number of Indoor Residual Spray (IRS) pump available for spray | Number of IRS pump available for spray |  | 100% | Annually |
|  |  |  |  |  |  |
|  | Proportion of Long-Lasting Insecticidal Nets (LLINs) received by district | Number of LLIN received by district | District Malaria Office (DMO) | - |  |
|  |  | Number of LLIN planned |  |  |  |
| **Process** | Proportion of Micro plans prepared for IRS | Number of micro plan prepared | DMO | - |  |
|  |  | Number of micro plan required |  |  |  |
|  | Proportion of Micro plans prepared for LLIN | Number of micro plan prepared | DMO | - |  |
|  |  | Number of micro plan required |  |  |  |
| **Output** | Proportion of eligible population covered by IRS | Total targeted population covered by IRS | Monitoring data | Year 2- 80%  Year 3- 90%  Year 4_100% | During IRS |
|  |  | Total targeted population for IRS |  |  |  |
|  | Targeted population covered by IRS  (This indicator will be segregated by children <5 years and pregnant females) | Total targeted population covered by IRS | Monitoring data | Year 2- 80%  Year 3- 90%  Year 4_100% | During IRS |
|  |  | Total targeted population for IRS |  |  |  |
|  | Proportion of targeted rooms covered by IRS | Total number of room sprayed | Monitoring data | Year 1-60%  Year 2-70%  Year 3- 80% | Half yearly |
|  |  | Total number of rooms targeted |  |  |  |
|  | Proportion of household owning LLIN | Number of households with LLIN | Monitoring data | Y1- >85% | Annually |
|  |  | Total number of households targeted for LLIN |  |  |  |
|  | Households (HH) with children < 5 years have LLIN | Total number of household with children <5 years have LLIN | Periodic assessment | Y1- >85% | Annually |
|  |  | Total number of household with children <5 years |  |  |  |
|  | Households with pregnant women having LLIN | Total number of household with pregnant have LLIN | Periodic assessment | Y1- >85% | Annually |
|  |  | Total number of household with pregnant female |  |  |  |
| **Outcome** | - - Beneficiaries (population under risk) slept under LLIN ** | Total number of household where member slept under LLIN | Periodic assessment | Year 1-60%  Year 2-70%  Year 3- 80% |  |
|  |  | Total number of household received LLIN |  |  |  |
|  | - - Proportion of children<5 years slept under LLIN | Total number of house hold where children<5 years slept under LLIN | Periodic assessment | Year 1-60%  Year 2-70%  Year 3- 80% |  |
|  |  | Total number of household with children<5 years received LLIN |  |  |  |
|  | - - Proportion of pregnant women sleeping under LLIN | Total number of household where pregnant women slept under LLIN | Periodic assessment | Year 1-60%  Year 2-70%  Year 3- 80% |  |
|  |  | Total number of household with pregnant women LLIN |  |  |  |
|  | Percentage of HHs accepting spraying as against surveyed | Number of households accepted spray | Monitoring data | Year 2- 80%  Year 3- 90%  Year 4_100% |  |
|  |  | Total number of HHs targeted for spray |  |  |  |
|  | Quality of spray operation | Number of HHs received quality spray | Monitoring data | >85% | Annually |
|  |  | Total number of HHS IRS checked |  |  |  |

**Key area 3:** Behaviour Change Communication (BCC) Strategy

| Level | Indicator | Definition | Data Source | Target | Frequency |
| --- | --- | --- | --- | --- | --- |
| Inputs | Proportion of funds available as against sanctioned  *(to be segregated for each activity under BCC)* | Funds available | Project |  | Monthly |
|  |  | Funds sanctioned |  |  |  |
|  | Proportion of health personnel in post as against sanctioned  *(to be segregated for every staff category under BCC)* | Number of health personnel in post | Project |  | Quarterly |
|  |  | Number of health personnel sanctioned |  |  |  |
|  | Proportion of School health leaders (SHL) identified | Number of School health leaders identified |  |  |  |
|  |  | Number of School health leaders planned |  |  |  |
|  | Proportion of Community leaders (CL) identified | Number of Community leaders identified | Planning calendar |  | Quarterly |
|  |  | Number of Community leaders planned |  |  |  |
|  | Proportion of Women health leaders (WHL) identified | Number of Women health leaders identified | Planning calendar |  | Quarterly |
|  |  | Number of Women health leaders planned |  |  |  |
| Process | Proportion of Haat bazar (community markers) coverage | Number of haat bazaar activities planned | Planning calendar |  | Quarterly |
|  |  | Number of targeted haat bazaars |  |  |  |
|  | Proportion of School level activities planned | Number of school level activities planned | Planning calendar |  | Quarterly |
|  |  | Number of targeted schools |  |  |  |
|  | Proportion of Blocks meetings planned | Number of block level activities planned | Planning calendar |  | Quarterly |
|  |  | Number of targeted block level meetings |  |  |  |
|  | Proportion of Self Help Group (SHG) meeting planned | Number of SHG meeting planned | Planning calendar |  | Quarterly |
|  |  | Number of targeted SHG meetings |  |  |  |
| Output | Haat bazaars attended in a Quarter | Number of haat bazaars level activities conducted | Monthly report | >80% | Quarterly |
|  |  | Number of haat bazaars where activities planned |  |  |  |
|  | School meetings attended | Number of school level activities conducted | Monthly report | >80% | Quarterly |
|  |  | Number of school where activities planned |  |  |  |
|  | Village Health, Sanitation and Nutrition Day (VHSND) attended | Number of VHSND level activities conducted | Monthly report | >80% | Quarterly |
|  |  | Number of VHSND where activities planned |  |  |  |
|  | % of SHL/WHL/CL disseminate information on malaria prevention and treatment | Number of SHL/WHL/CL who disseminate information on malaria | Periodic assessment forms | >80% | Quarterly |
|  |  | Number of SHL/WHL/CL who were identified & trained (oriented) |  |  |  |
| Outcome | Awareness about LLIN use | Number of interviewed people use of LLIN | Periodic assessment forms | >80% | Half yearly |
|  |  | Number of people interviewed |  |  |  |
|  | Awareness about prevention of malaria | Number of interviewed people know various method to prevent malaria | Periodic assessment forms | >80% | Half yearly |
|  |  | Number of people interviewed |  |  |  |
|  | Awareness about govt. health facilities | Number of people has knowledge of govt. facility where malaria it is available | Periodic assessment forms | >80% | Half yearly |
|  |  | Number of people interviewed |  |  |  |
|  | Health seeking behaviour | Number of fever cases who visited govt. facilities | PA forms | >80% | Half yearly |
|  |  | Number of fever cases (History) interviewed |  |  |  |

Note:

Indicators with ** are common in GTS and NFME, Indicators with * are only in GTS, Indicators with # are only in NFME.
